# Budesonide and formoterol effects on rhinovirus replication and epithelial cell cytokine responses

**DOI:** 10.1186/1465-9921-14-98

**Published:** 2013-10-04

**Authors:** Yury A Bochkov, William W Busse, Rebecca A Brockman-Schneider, Michael D Evans, Nizar N Jarjour, Christopher McCrae, Anna Miller-Larsson, James E Gern

**Affiliations:** 1 Department of Pediatrics, University of Wisconsin-Madison, 600 Highland Avenue, K4/945 CSC, Madison, WI 53792-9988, USA; 2 Department of Medicine, University of Wisconsin-Madison, Madison, WI, USA; 3 Department of Bioinformatics, University of Wisconsin-Madison, Madison, WI, USA; 4 AstraZeneca R&D Mölndal, Mölndal, Sweden

**Keywords:** Budesonide, Formoterol, Human rhinovirus, Bronchial epithelial cells, Asthma

## Abstract

**Background:**

Combination therapy with budesonide and formoterol reduces exacerbations of asthma, which are closely associated with human rhinovirus (RV) infections in both children and adults. These data suggest that budesonide and formoterol inhibit virus-induced inflammatory responses of airway epithelial cells.

**Methods:**

To test this hypothesis, bronchial epithelial (BE) cells were obtained from airway brushings of 8 subjects with moderate-to-severe allergic asthma and 9 with neither asthma nor respiratory allergies. Cultured BE cells were incubated for 24 hours with budesonide (1.77 μM), formoterol (0.1 μM), both, or neither, and then inoculated with RV-16 (5×10^6^ plaque forming units [PFU]/mL). After 24 hours, viral replication (RV RNA), cytokine secretion (CXCL8, CXCL10, TNFα, IFN-β, IL-28) and mRNA expression (CXCL8, CXCL10, TNF, IFNB1, IL28A&B) were analyzed.

**Results:**

RV infection induced CXCL10 protein secretion and IFNB1 and IL28 mRNA expression. Drug treatments significantly inhibited secretion of CXCL10 in mock-infected, but not RV-infected, BE cells, and inhibited secretion of TNFα under both conditions. Neither budesonide nor formoterol, alone or in combination, significantly affected viral replication, nor did they inhibit RV-induced upregulation of IFNB1 and IL28 mRNA. Overall, RV replication was positively related to CXCL10 secretion and induction of IFNB1 and IL28 mRNA, but the positive relationship between RV RNA and CXCL10 secretion was stronger in normal subjects than in subjects with asthma.

**Conclusions:**

Budesonide and formoterol can inhibit BE cell inflammatory responses *in vitro* without interfering with viral replication or production of interferons. These effects could potentially contribute to beneficial effects of budesonide/formoterol combination therapy in preventing RV-induced asthma exacerbations.

## Background

Infections with human rhinoviruses (RVs) are commonly associated with increased symptoms and acute exacerbations of asthma that require treatment in the emergency department or hospitalization. In fact, RV infections are more often detected during acute exacerbations than any other respiratory virus [1,2]. This close association has led to research efforts directed at identifying treatment to prevent RV-induced exacerbations of asthma.

Several medications have shown efficacy for prevention of asthma exacerbations in general. Combination therapy with an inhaled corticosteroid (ICS) together with long-acting beta agonist (LABA) is particularly effective at preventing exacerbations [3,4]. Results of clinical studies also suggest that both ICS and combination medications prevent exacerbations during the common cold season, and exacerbations that are associated with symptoms of the common cold [5,6].

Defining mechanisms of these effects is important to improving the efficacy of medications that prevent exacerbations, and several mechanisms have been proposed. First, RVs initiate infections by first binding to cell surface receptors on airway epithelial cells. Notably, ICS can downregulate *in vitro* expression of intercellular adhesion molecule 1 (ICAM-1), which is the cellular receptor for 89 of the 100 canonical RV-A and RV-B types [7,8]; however, glucocorticoid treatment did not prevent the increased ICAM-1 expression after experimental RV-16 infection [9]. Second, RVs cause illnesses in part by inducing release of pro-inflammatory chemokines and cytokines from infected epithelial cells. These factors include chemoattractants for neutrophils (e.g. CXCL8), mononuclear cells and T cells (e.g. CXCL10), and cytokines such as TNFα that can potentiate inflammatory cell functions [10-13]. ICS can inhibit the RV-induced production of both pro-inflammatory mediators [14] and type I interferons (e.g. IFN-α) that have antiviral effects [15]. Clinical studies have shown evidence of this effect *in vivo* in that either systemic or topical corticosteroids were associated with increased RV replication after experimental inoculation with virus; however, cold severity was not affected [16].

We hypothesized that the combination of budesonide and formoterol would inhibit virus-induced inflammatory responses of primary airway epithelial cells *in vitro*. To test this hypothesis, we conducted a series of experiments with bronchial epithelial (BE) cells that were obtained from subjects with or without allergic asthma. As a secondary objective, we compared viral replication and RV-induced responses in cells from subjects with and without allergic asthma.

## Methods

### Study subjects

Primary BE cells were obtained from bronchial brushings of normal subjects and those with moderate to severe allergic asthma (Table 1, *n* = 17). The subjects with allergic asthma were treated with inhaled or oral corticosteroids, and these medications were continued up until the time of bronchoscopy for reasons of safety. The study protocol was approved by the University of Wisconsin-Madison Human Subjects Committee. After obtaining informed consent, lung function tests and methacholine responsiveness were determined as described previously [17]. Skin testing was performed to detect IgE specific for aeroallergens of local importance in Madison, WI [17]. In the asthma group, all subjects had PC_20_ <8 mg/mL or >12% reversibility in FEV_1_ after bronchodilator, and all had positive skin tests for aeroallergen-specific IgE. In the normal group, lung function tests were normal, and there was no evidence of either bronchial hyperresponsiveness or allergic sensitization.

**Table 1:** Study Subjects

**Subject**	**Group**	**Gender**	**Age**	**PC**_**20 **_**(mg/mL)**	**FEV1 % predicted**	Reversibility* (%)	**Allergen Skin Test**
1	N	M	24	20	86	7	neg
2	N	M	21	20	99	5	neg
3	N	M	22	50	101	7	neg
4	N	M	21	50	90	5	neg
5	N	F	20	50	86	6	neg
6	N	M	40	50	101	1	neg
7	N	M	30	50	106	4	neg
8	N	F	24	50	99	3	neg
9	N	F	21	16	102	6	neg
Mean				36	97	5	
10	A	F	20	4.2	101	6	pos
11	A	F	20	0.4	98	-2	pos
12	A	M	52	nd	48	22	pos
13	A	F	36	0.3	64	51	pos
14	A	M	46	nd	62	14	pos
15	A	F	24	0.9	94	4	pos
16	A	M	27	0.3	55	27	pos
17	A	M	21	0.8	80	15	pos
Mean				0.7	75	17	

*Definition of abbreviations*: N, normal; A, asthma; nd, not done; PC_20_, provocative concentration of methacholine causing a 20% fall in FEV1; FEV1, forced expiratory volume in one second.

*Change in FEV1 after inhaling 2 puffs of albuterol.

### Bronchial epithelial cells

BE cells from bronchial brushings were cultured as described previously [11,18]. Briefly, the cells were removed from the brush by scraping and vigorous agitation in Dulbecco's Modified Eagle Medium: Nutrient Mixture F-12 (DMEM/F12, Life Technologies, Grand Island, NY), and then placed in tissue culture in collagen coated plasticware at 37°C (5% CO_2_) in Bronchial Epithelial Growth Medium (BEGM, Lonza, Walkersville, MD) in 25 cm^2^ flasks (passage 0), then expanded in 75 cm^2^ flasks (passage 1), and finally seeded in 12-well plates (passage 2). Purified and concentrated RV-16 [19] was diluted in BEGM with a reduced concentration (10^-8^ M) of hydrocortisone (low Hc BEGM) just before infection. Stock solutions of budesonide (anhydrous) and formoterol fumarate (dihydrate) were prepared in DMSO at a concentration of 10 mM, subsequent dilutions were made in low Hc BEGM (final concentration of DMSO applied to cells was ≤ 0.01%). When the cell monolayers (duplicate wells) from each study subject (passage 2) were approximately 70-80% confluent, they were pre-treated for 24 h with budesonide (1.77 μM), formoterol (0.1 μM), both (at recommended therapeutic ratio of 17.7:1), or neither (0.01% DMSO drug vehicle), and then either infected with 0.25 mL of RV-16 (5×10^6^ plaque forming units [PFU]/mL), or mock-infected with medium alone for 2 h at 34°C. Cell monolayers were then washed three times with PBS before adding fresh low Hc BEGM. After 24 h incubation at 34°C (5% CO_2_), cell supernatants were collected, the monolayers were washed with phosphate buffered saline and lysed by adding TRIzol Reagent (Invitrogen, Carlsbad, CA). Supernatant and cell lysate samples were stored in microcentrifuge tubes at -80°C until RNA extraction and cytokine analysis.

### Virology

The major receptor group RV-16 was chosen for this study because major group viruses replicate well in cultured BE cells without causing marked cytopathic effects [11], and thus is a good model of *in vivo* infections [20]. Stocks of virus were grown in HeLa cells and purified by sucrose gradient centrifugation as described previously [21].

### Measurement of viral and cellular RNAs

Total RNA was isolated from the frozen TRIzol lysates according to manufacturer’s protocol, treated with RQ1 DNase (Promega, WI, USA) and then purified by the RNeasy Mini Kit (Qiagen, Hilden, Germany). First-strand cDNA synthesis was performed using the TaqMan Reverse Transcription reagents and random hexamers (Life Technologies, Grand Island, NY). PCR was performed as previously described [18] using the primers shown in Table 2. *Power* SYBR Green PCR master mix (Life Technologies) was used to perform the reactions. Fold differences were determined by the 2^–∆∆Ct^ method. The cycle threshold (Ct) of each transcript was normalized to the Ct of the house-keeping gene peptidylprolyl isomerase A (PPIA) [22]. IL28 primers detect both IL28A and IL28B mRNAs due to their high sequence identity. We used RV primers and probe as described previously [20]. A standard curve was derived from purified RV-16 prep of known infectivity, and results are reported in PFU equivalents (PFUe).

**Table 2:** Real-time PCR primers used for mRNA quantification

**Target gene**	**Sequence( 5’-3’)**	**Primer length**	**Amplicon size (bp)**
PPIA-f^†^	TCATCTGCACTGCCAAGACTG	21	
PPIA-r^†^	CATGCCTTCTTTCACTTTGCC	21	71
IFNB1-f	AGGACGCCGCATTGACC	17	
IFNB1-r	ATTCCAGCCAGTGCTAGATGA	21	83
IL28-f	GCTGACCGTGACTGGAGCA	19	
IL28-r	GACAGGGACTTGAACTGGGCTA	22	93
TNF-f	TCGGCCCGACTATCTCGAC	19	
TNF-r	GCGTTTGGGAAGGTTGGAT	19	94
CXCL10-f	AAGCTCTACTGAGGTGCTATGTT	23	
CXCL10-r	TGGGAAAGGTGAGGGAAATA	20	72
CXCL8-f	CAGTTTTGCCAAGGAGTGCTAA	22	
CXCL8-r	GGTGGAAAGGTTTGGAGTATGTC	23	69

*Definition of abbreviations*: f, forward primer; r, reverse primer; bp, base pairs.

^†^The PPIA primer sequences were published by RTPrimerDB, ID #3460 (http://medgen.ugent.be/rtprimerdb/).

### Measurement of cytokine proteins

CXCL8, CXCL10, TNFα and IL-28 concentrations were assessed using human Cytokine / Chemokine Milliplex map kits (Millipore, Temecula, CA) according to the manufacturer’s instructions. Luminex® 100 instrument (Luminex Corporation, Austin, TX) was used to run plates and generate quantitative data. Sensitivity of the assays (minimum detectable concentration) was 0.3 pg/mL (CXCL8), 2.2 pg/mL (CXCL10), 7.9 pg/mL (IL-28) and 0.1 pg/mL (TNFα). IFN-β secretion was determined by ELISA (R&D Systems, Minneapolis, MN) according to the manufacturer’s instructions. The lower limit of IFN-β detection was 50 pg/mL.

### Data analysis

Mixed-effects ANOVA models were used for all analyses to account for repeated measures in subjects. All responses were log-transformed for analysis except CXCL8 protein. Means and confidence intervals (CIs) of log-transformed responses were back-transformed and reported on the original scale of measurement. In the absence of significant interaction effects, the main effects for asthma status, RV versus mock treatment, and drug, are reported. Associations between viral RNA and cytokine mRNA and protein levels were tested in linear models of CXCL10 with RV, asthma, and RV × asthma interaction as covariates. Pearson correlation coefficients were calculated for relationships between RV and cytokine mRNA and protein. P values < 0.05 were considered statistically significant.

## Results

### Viral replication

**Figure 1:**
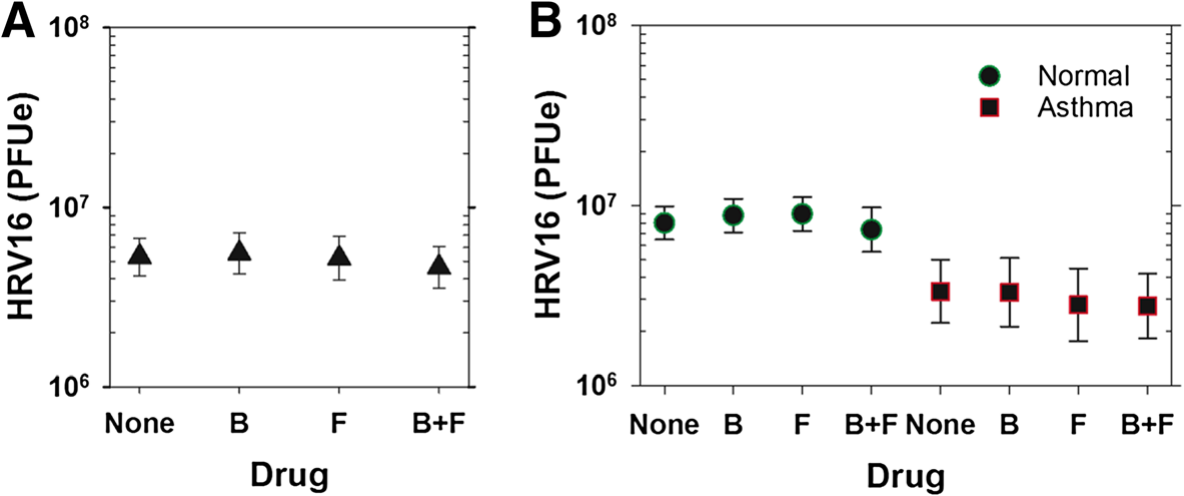
**Viral replication in BE cells from normal and asthmatic subjects was not affected by budesonide or formoterol.** Cells were inoculated with RV-16 after 24 h pre-treatment with budesonide (B), formoterol (F), both drugs (B+F), or media alone (none) and cell-associated viral RNA was measured 24 h post-inoculation. Neither B, F, nor the combination significantly affected RV replication in all samples **(*A*)**, nor in samples grouped according to asthma status **(*B*)**. Data are represented as geometric means with 95% confidence intervals.

Measurements were performed in cell lysates (RV RNA, cytokine mRNA) and supernatants (cytokine protein) collected 24 h post inoculation. When all subjects (*n* = 17) were considered together, the geometric mean of RV-16 RNA was 5.3 × 10^6^ (3.2 × 10^6^, 8.8 × 10^6^, 95% CI) PFUe per sample in the absence of drug treatment (Figure 1A). Treatment with budesonide and/or formoterol had no effect on RV replication (Figure 1A, *p* = 0.43). We next examined effects of allergic asthma on viral replication and induction of cytokines in ANOVA models including subject, asthma status, and drug treatment group. Overall, viral replication tended to be greater in cells from normal subjects compared to cells from subjects with asthma (7.6 ×10^6^ [4.1 × 10^6^, 14.2 × 10^6^] vs. 3.3 × 10^6^ [1.7 × 10^6^, 3.3 × 10^6^] PFUe, *p* = 0.06; Figure 1B).

### Drug effects on cytokine induction

**Figure 2:**
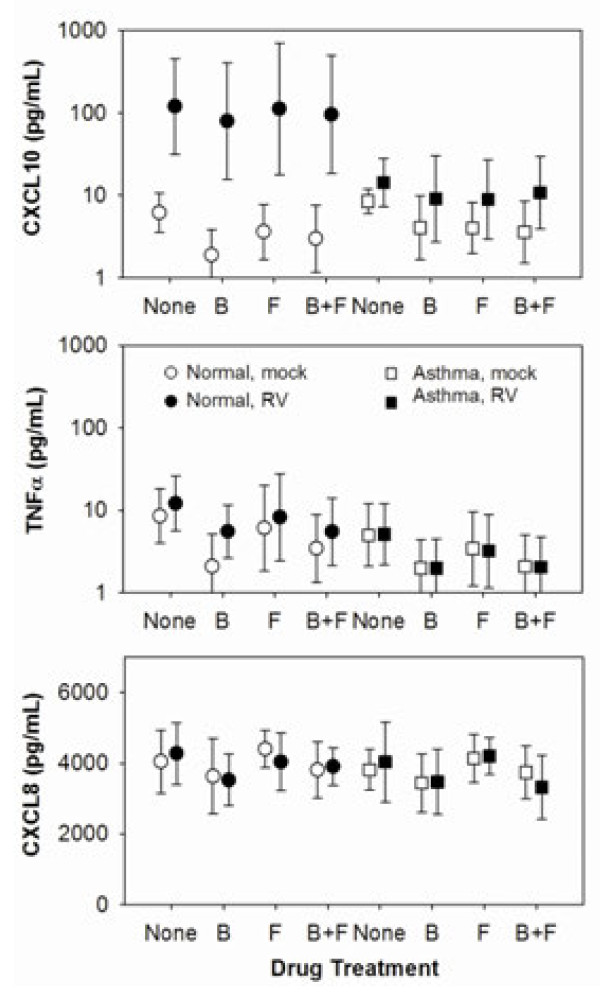
**Effects of RV infection and drug treatments on cytokine protein secretion by BE cells from normal and asthmatic subjects.** Cells were inoculated with RV-16 after 24 h pre-treatment with budesonide (B), formoterol (F), the combination (B+F) or media alone (none), and samples of tissue culture medium obtained 24 h post inoculation were tested for cytokines. Data are represented as geometric (CXCL10, TNFα) or arithmetic (CXCL8) means with 95% confidence intervals.

**Figure 3:**
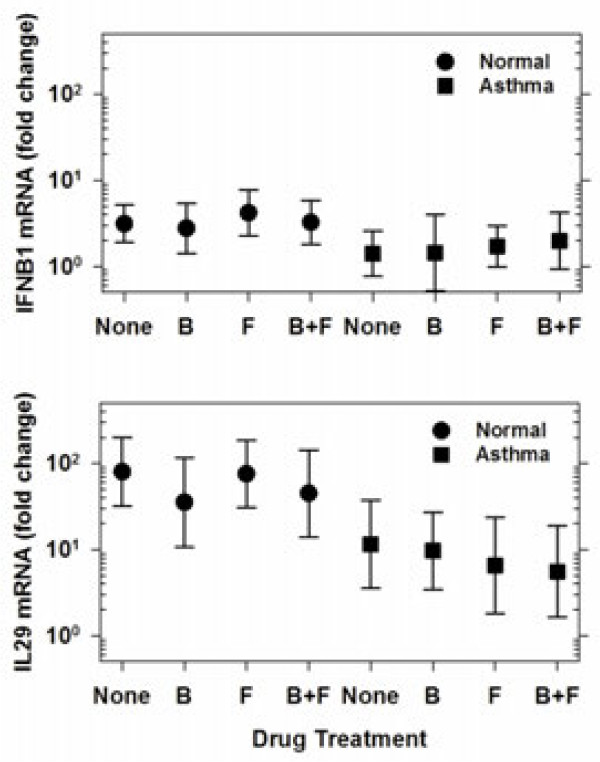
**Treatment effects on expression of interferon mRNA by BE cells from normal and asthmatic subjects.** Cells were inoculated with RV-16 after 24 h pre-treatment with budesonide (B), formoterol (F), the combination (B+F) or media alone (none) and then type I (IFNB1) and III (IL28) interferon mRNA levels were measured by qPCR in cell lysates 24 h post inoculation. Data are represented as geometric mean fold change compared to mock-infected cells with 95% confidence intervals.

RV-16 infection significantly induced secretion of CXCL10 (44 [7.0, 249] vs. 7.2 [5.3, 9.8] pg/mL geometric means [95% CI], *p* < 0.001), but not TNFα or CXCL8 (Figure 2). We also tested for IL-28 and IFN-β secretion, but these cytokines were not detectable in most samples, and we therefore analyzed IL28 and IFNB1 mRNA expression by qPCR. Compared to mock-infected cells and in the absence of drug treatment, RV induced small amounts of IFNB1 mRNA (2.2 [1.4, 2.2] fold increase) and larger amounts of IL28 mRNA (32.2 [14.1, 73.6] fold increase) (Figure 3), but did not induce TNF or CXCL8 mRNA (data not shown).

In mock-infected cells, treatment with budesonide, formoterol, and the combination of both drugs significantly inhibited secretion of CXCL10 and TNFα compared to cells without drug treatment (Table 3). None of the drug treatments had significant effects on CXCL8 secretion in mock-infected cells.

**Table 3:** **Effects of drugs on cytokine secretion by BE cells from all subjects (mean**^**†**^**, 95% CI, *n *= 17)**

**Mock-infected**	**Drugs**			
	**None**	**Budesonide**	**Formoterol**	**Both**
CXCL8	3940 (3454, 4426)	3546 (2935, 4157)	4279 (3901, 4657)	3780 (3292, 4268)
CXCL10	7.2 (5.3, 9.8)	2.7 (1.6, 4.6)**	3.8 (2.4, 6.1)**	3.3 (1.8, 5.8)**
TNFα	6.7 (3.9, 11)	0.5 (0.2, 1.4)**	2.4 (0.7, 7.8)*	0.8 (0.2, 2.6)**
**RV-infected**				
CXCL8	4163 (3540, 4786)	3504 (2993, 4015)**	4116 (3675, 4557)	3634 (3164, 4104)*
CXCL10	44 (7.0, 249)	29 (9.8, 84)	34 (11, 112)	34 (12, 99)
TNFα	8.1 (4.7, 14)	1.5 (0.5, 4.6)**	2.7 (0.8, 9.2)**	1.3 (0.4, 4.4)**
IFNBI mRNA	2.2 (1.4, 3.2)	2.0 (1.2, 3.8)	2.8 (1.8, 4.3)	2.6 (1.7, 4.0)
IL28 mRNA	32 (14, 74)	19 (8.9, 42)	24 (9.4, 61)	17 (6.7, 42)

^†^CXCL8 arithmetic mean, others represent geometric means; cytokine protein values represent pg/mL, and mRNA are presented as fold increase after RV infection.

* P ≤ 0.05 drug vs. no drug.

** P ≤ 0.005 drug vs. no drug.

In RV-infected samples, treatment with both drugs alone and the combination inhibited secretion of TNFα, but effects on CXCL10 were not significant (Table 3). Treatment with either budesonide or the combination of budesonide and formoterol also inhibited CXCL8 secretion after RV infection. There were no significant treatment effects of budesonide, formoterol, or the combination on RV-induced IFNB1 or IL28 mRNAs (*p* = 0.27 and 0.14 respectively, Table 3).

### Asthma effects on cytokine induction

Asthma appeared to modify RV-induced secretion of CXCL10 and TNFα (Table 4) in that RV infection stimulated increased secretion of these cytokines only in subjects without asthma (asthma × RV interaction *p* values: CXCL10, 0.003; TNFα, 0.0003). Similarly, asthma was associated with lower fold increases in RV-induced IFNB1 mRNA (*p* = 0.01) and IL28 mRNA (*p* = 0.009). There were no effects of asthma on secretion of CXCL8 (data not shown).

**Table 4:** Effects of asthma status on baseline (Mock) and RV-induced cytokine secretion and interferon mRNA expression (geometric mean, 95% CI)

**Cytokine**		**Normal**	**Asthma**	**P value (asthma vs. normal)**
**CXCL10 (pg/mL)**	Mock	3.4 (2.2, 5.1)	5.3 (3.5, 8.2)	0.06
	RV	50.6 (21.6, 118.1)**	10.1 (4.2, 24.4)	0.003
**TNFα (pg/mL)**	Mock	3.2 (1.3, 7.9)	1.4 (0.6, 3.5)	0.02
	RV	4.7 (1.9, 11.3)*	1.4 (0,6, 3.5)	0.0003
**IFNB1 mRNA (fold increase)**		3.4 (2.2, 5.3)	1.5 (1.0, 2.5)	0.01
**IL28 mRNA (fold increase)**		49 (21, 112)	9.2 (3.8, 22)	0.009

*P = 0.006, ** P < 0.0001 for RV vs. mock-infected samples.

**Figure 4:**
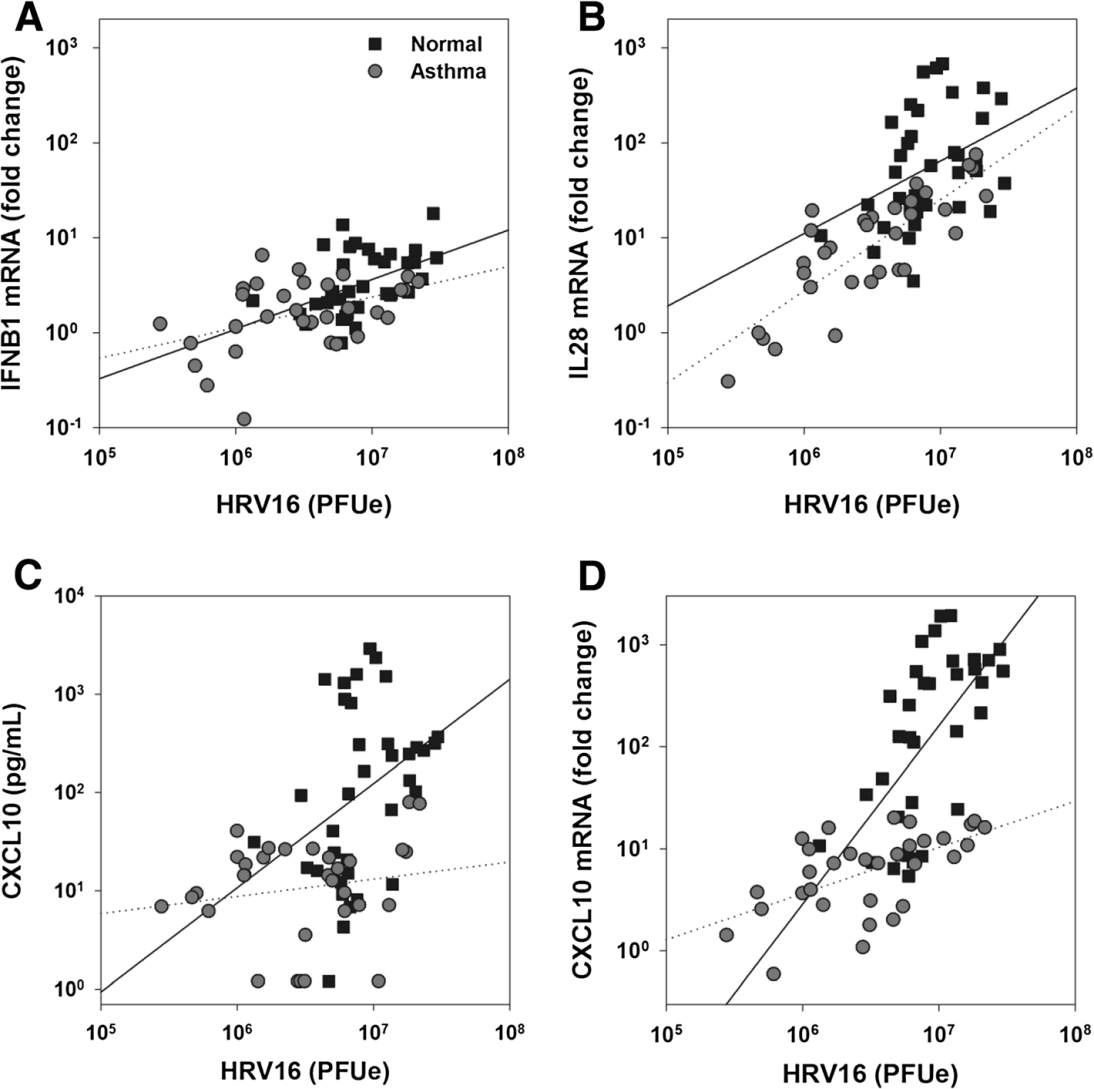
**Correlations between RV RNA levels and cytokine responses in BE cells.** RV RNA 24 h after inoculation was positively associated with IFNB1 mRNA **(*A*)**, IL28 mRNA **(*B*)**, and CXCL10 cytokine secretion **(*C*)** and mRNA upregulation **(*D*)**. Samples include untreated samples and also those treated with budesonide, formoterol, and the combination. Results from samples in the normal and asthma groups are indicated by squares and circles, respectively, and separate regression lines (solid line, normal; dotted line, asthma) are plotted for each group.

Given the trend for less viral replication in the samples from subjects with asthma, we conducted a post hoc analysis to determine whether asthma status affected RV induction of cytokine protein and mRNA. Overall, there was a positive correlation between RV RNA levels and induction of both IFNB1 mRNA (*r* = 0.69, *p* < 0.001) and IL28 mRNA (*r* = 0.71, *p* < 0.001), and this relationship was not affected by asthma status (Figures 4A and 4B; interaction terms *p* = 0.36 for IFNB1 and *p* = 0.54 for IL28). In contrast, there was a positive correlation between RV RNA level and CXCL10 secretion in cells from normal individuals (*r* = 0.36, *p* = 0.01), but not in cells from subjects with asthma (*r* = 0.17, *p* = 0.48; interaction term p=0.07; Figure 4C). For example, a 10-fold increase in RV RNA was associated with a 11-fold increase (95% CI 1.9, 70) in CXCL10 secretion in the normal group, compared to only a 1.5 fold increase (95% CI 0.49, 4.6) in the asthma group.

To determine whether this difference was related to measurement of protein vs. mRNA, we also compared RV RNA to induction of CXCL10 mRNA. RV RNA was positively correlated with CXCL10 mRNA in both groups (no asthma *r* = 0.61, *p* < 0.0001; asthma *r* = 0.57, *p* < 0.0001), but the slope was significantly greater in the no asthma group (interaction term *p* = 0.0008; Figure 4D). In this case, a 10-fold increase in RV RNA was associated with a 56-fold increase (95% CI 14, 230) in CXCL10 mRNA in the normal group, compared to a 2.8-fold increase (95% CI 1.2, 6.8) in CXCL10 mRNA in the asthma group.

## Discussion

In addition to serving as host cells for viral infections, airway epithelial cells are important initiators of the local antiviral immune response through the production of chemokines, pro-inflammatory cytokines, and interferons [23]. These studies were conducted to determine the effects of budesonide, formoterol and the combination of these two drugs on RV-induced cytokine responses of cultured primary BE cells. Because primary cells obtained by bronchoscopy are usually low in number and have limited growth potential, we have used high but still clinically relevant concentration of drugs that had shown strong inhibitory effects *in vitro*[24-26] instead of testing multiple drug concentrations. The results show that RV infection stimulated CXCL10 secretion and an antiviral response as indicated by induction of IFNB1 and IL28 mRNA. Secretion of CXCL10 was significantly inhibited by budesonide, formoterol and their combination in mock-infected cells, but none of the treatments significantly affected RV-16-induced CXCL10 secretion. Notably, budesonide, formoterol or their combination did not significantly affect antiviral responses as measured by IFNB1 and IL28 mRNA expression, nor did they significantly affect viral replication. In contrast, treatment with these drugs inhibited TNFα secretion with or without infection, and budesonide or combination of budesonide and formoterol inhibited CXCL8 secretion after infection. When considered together, these findings suggest that budesonide and formoterol hinder BE cell secretion of specific pro-inflammatory cytokines without affecting epithelial antiviral responses.

Another goal of these studies was to test for allergic asthma effects on epithelial cell immune responses to RV. In cells from normal individuals, RV infection induced secretion of CXCL10 and TNFα; these responses were significantly blunted in the asthma group. Furthermore, although levels of interferon protein secretion were undetectable in most cultures, viral infection consistently upregulated IFNB1 and IL28 mRNA, and these responses were also lower in the asthma group. Some [27,28], but not all [17,18,29], previous studies have suggested that epithelial cell interferon responses are lower in BE cells from subjects with asthma, and these low interferon responses were inversely related to viral replication. In contrast, interferon responses were positively related to viral replication in our study, and the lower interferon responses observed in the asthma group were associated with a trend for lower viral replication. Although the reason for lower replication in the asthma group is unclear, we speculate that use of inhaled or oral corticosteroids by the subjects with asthma could have suppressed ICAM-1 expression, and that this suppression may have persisted through several cell generations in tissue culture. Interestingly, post hoc analyses suggest that allergic asthma status also affected the relationship between viral replication and induction of CXCL10, a chemokine for Th1 cells and mononuclear cells that is strongly induced by RV infection both *in vitro* and *in vivo*[10,17,30]. In our study, the positive correlation between RV RNA and CXCL10 mRNA and protein was blunted in subjects with allergic asthma (Figure 4C and 4D). Additional studies are warranted to determine whether this effect is also present *in vivo*.

Budesonide and formoterol combination therapy has been used extensively in the treatment of asthma, and controls daily symptoms as well as reducing the risk for asthma exacerbations [4]. The fact that most asthma exacerbations in children and about half of those in adults have viral infection as a contributing factor [31] suggests that combination therapy may specifically inhibit virus-induced exacerbations. Our findings as well as some previously published *in vitro* results [24,25] suggest that budesonide and formoterol-mediated reduction of BE cell inflammatory responses may contribute to this effect. Skevaki et al. [25] have shown that the combination of budesonide and formoterol administered post infection suppressed RV-induced pro-inflammatory cytokines (CCL5, CXCL8 and CXCL10) and the remodeling-associated growth factor (VEGF) in BE cells in a synergistic or additive manner. In our experimental model, pre-treatment with budesonide alone or in combination with formoterol also inhibited pro-inflammatory cytokines (CXCL10, TNFα and CXCL8) at baseline and/or after RV infection. Synergistic or additive suppression of RV-induced chemokines was also observed after pre-treatment of cultured BE cells with fluticasone propionate and salmeterol [14]. In RV-stimulated peripheral blood mononuclear cells, the combination of budesonide and formoterol inhibited CXCL10 and also antiviral responses (IFN-α secretion and expression of the type I interferon inducible genes) [15]. In contrast, we did not observe significant inhibitory effects of the drugs on RV-induced upregulation of CXCL10 secretion or type I and III interferon genes (e.g. IFNB1 and IL28) in primary BE cells. Interestingly, RV-16 infection has been shown to reduce glucocorticoid sensitivity of airway epithelial cells *in vitro* through activation of c-Jun N-terminal kinase (JNK) and nuclear factor κB (NF-κB) signaling pathways; however, potential effects of combined treatment with beta agonists have not been studied [32].

This study has several strengths, and some limitations, that should be considered in interpreting these results. Strengths of the study include the use of primary cultures of BE cells, as well as including cells from donors who were characterized for allergy and asthma. The donors had moderate or severe allergic asthma with well-characterized abnormalities in lung function. Furthermore, we used highly purified preparations of RV-16, as well as sensitive and specific viral diagnostics (real-time RT-PCR) for these studies. One limitation of the study is that the asthma and no asthma groups also differed in allergic status, and so some of the differences attributed to asthma could be mediated by allergy. In addition, each of the subjects with asthma was treated with inhaled or oral corticosteroids, and these medications were continued up until the time of bronchoscopy for reasons of safety. Although effects of the corticosteroid preparations may have been partially washed out under the conditions in tissue culture, pre-study treatment could have influenced findings *in vitro*. These *in vitro* studies enabled us to isolate responses of BE cells to virus and drugs, however, it is obvious that epithelial cell responses to viral infection as well as viral clearance are affected by other cells and cytokines in the airway milieu [33,34], and correlation with clinical findings is needed to establish relevance *in vivo*. It has been shown that the proportion of RV-16-infectible airway epithelial cells is relatively low (less 10%) in both monolayer cultures and bronchial tissue samples infected *ex vivo* primarily due to a low basal expression of ICAM-1, the receptor for RV-16 and other major group viruses [35]. Therefore, the modest magnitude of virus-specific cytokine induction in these experiments was likely due to restriction of infection to the small subset of BE cells that express ICAM-1.

## Conclusions

In summary, these findings indicate that budesonide and formoterol can inhibit inflammatory responses *in vitro* in primary BE cells without affecting viral replication or interfering with induction of the antiviral genes IFNB1 and IL28. These effects could potentially contribute to beneficial effects of budesonide and budesonide/formoterol combination therapy in preventing RV-induced asthma exacerbations *in vivo*. Furthermore, our results raise additional questions about the etiology and potential clinical significance of asthma-related differences in BE cell responses to RV infection.

## Competing interests

This work was supported by investigator-initiated research grant from AstraZeneca. YAB, RAB and MDE declare that they have no competing interests. WWB provides advisory board services to Merck; consulting services to Amgen, Novartis, GlaxoSmithKline, MedImmune, and Genentech; is a member of Data Monitoring Boards and Study Oversight Committees for Boston Scientific, Genentech, and ICON; receives royalties from Elsevier, and receives NIH grant support from NIH-NIAID and NIH-NHLBI. CM and AML are employees of AstraZeneca. JEG has grant funding from GlaxoSmithKline and Merck and is a consultant to AstraZeneca, GlaxoSmithKline, Merck, Gilead, Johnson & Johnson, and Boehringer Ingelheim.

## Authors’ contributions

YAB participated in the study design, performed experiments, analyzed data and drafted the manuscript. WWB and NNJ participated in the study design and interpretation of data. RAB assisted with cell culture. MDE contributed to data analysis. CM and AML participated in data analysis and writing of the manuscript. JEG designed the project, analyzed data and was the senior author of the paper. All authors read and approved the final manuscript.
